# Left main trunk stenting in a case of acute aortic dissection: a case report

**DOI:** 10.1002/ccr3.1164

**Published:** 2017-08-29

**Authors:** Masaomi Gohbara, Tsutomu Endo, Kazuo Kimura, Kouichi Tamura

**Affiliations:** ^1^ Division of Cardiology Saiseikai Yokohamashi Nanbu Hospital Yokohama Japan; ^2^ Division of Cardiology Yokohama City University Medical Center Yokohama Japan; ^3^ Department of Medical Science and Cardiorenal Medicine Yokohama City University Graduate School of Medicine Yokohama Japan

**Keywords:** Aortic dissection, myocardial infarction, percutaneous coronary intervention

## Abstract

In cases involving unstable hemodynamics in patients with Stanford type‐A acute aortic dissection involving left main trunk (LMT) compression, LMT stenting without antiplatelet agents may be a good option as a bridge to surgery.

## Background

Stanford type‐A acute aortic dissection (AAD) is a life‐threatening disorder with a high mortality rate of 48.6% before hospital assessment in the previous population‐based study 1. It sometimes causes left main trunk (LMT) compression by the false lumen, leading to broad anterolateral myocardial ischemia and resulting in a life‐threatening status 2. It is important to take the possibility of Stanford type‐A AAD into consideration upon recognizing broad anterolateral myocardial ischemia by electrocardiography (ECG) or echocardiography. A previous report revealed that the rate of the malperfusion of the coronary artery was 6.8% in patients with Stanford type‐A AAD 3. Stent implantation into the LMT ostium in patients with Stanford type‐A AAD involving LMT compression is one method as a bridge to surgery 4; however, there is no report that discusses LMT stenting without antiplatelet agents to reduce the risk of perioperative bleeding.

## Case Presentation

**Figure 1:**
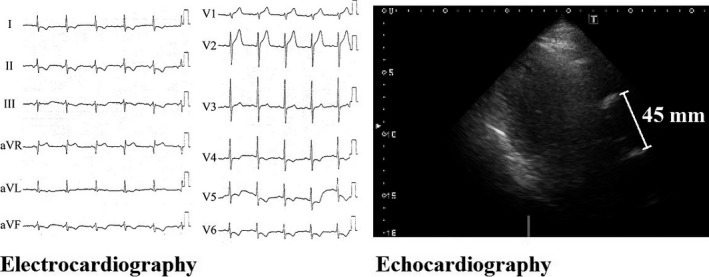
Electrocardiography and echocardiography findings. Admission electrocardiography revealed normal sinus rhythm with narrow QRS duration, ST‐segment elevation in lead aVR, and ST‐segment depression in leads I, II, III, aVL, aVF, and V4‐6. Echocardiography also revealed broad anterolateral akinesis without aortic regurgitation, pericardial effusion, or definite aortic flap. The diameter of the aortic root was 45 mm.

**Figure 2:**
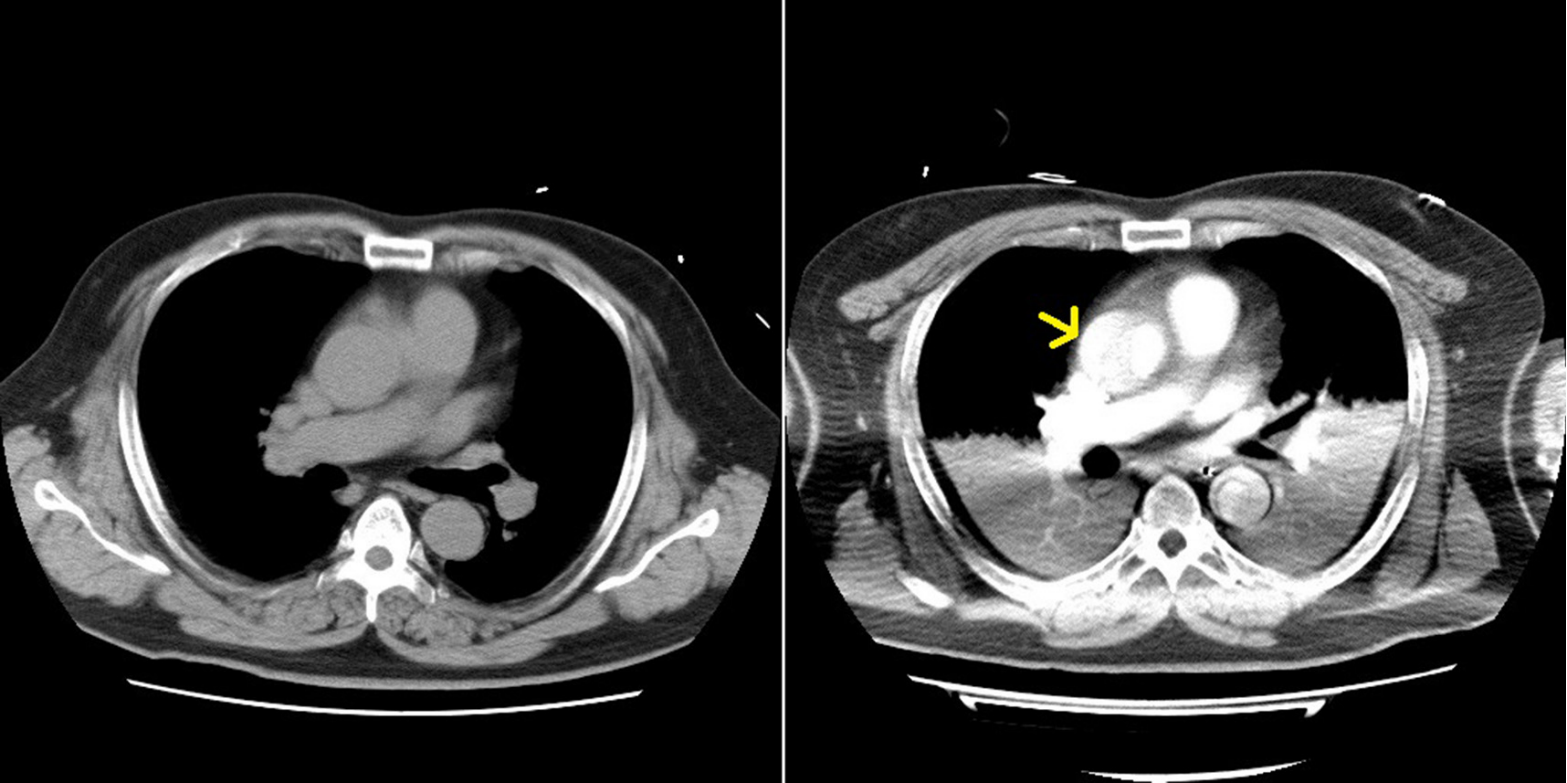
Computed tomography imaging. Plain computed tomography (CT) imaging demonstrated no definite AAD (on left). Contrast‐enhanced CT imaging after PCI demonstrated Stanford type‐A AAD (on right; a yellow arrow points out the false lumen of the ascending aorta). AAD, acute aortic dissection; PCI, percutaneous coronary intervention.

**Figure 3:**
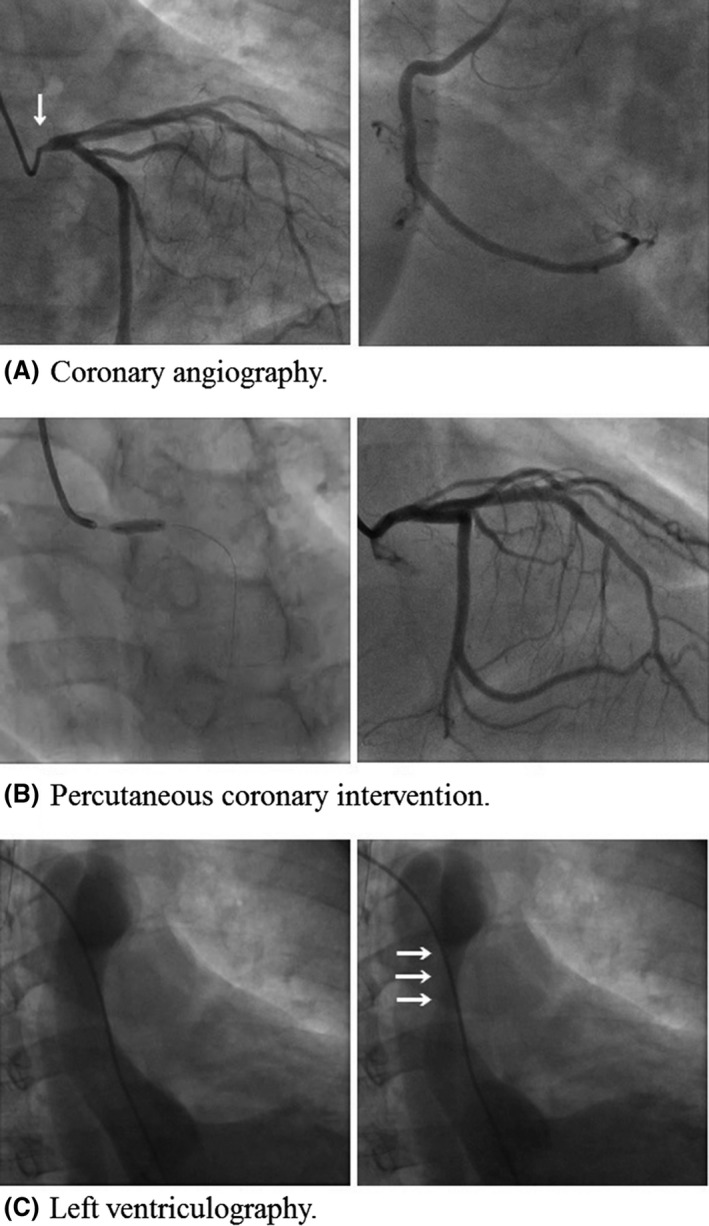
Coronary angiography and left ventriculography. (A) The left figure demonstrates the left coronary artery, and the right figure demonstrates the right coronary artery. Seventy‐five percent stenosis of LMT ostium was observed, as shown in the left figure (via a white arrow). (B) A bare‐metal stent (3.5 mm × 9 mm) was implanted into the LMT ostium and 2–3 mm into the aorta as a bridge to surgery with low pressure, so that a surgeon could pull out the stent during surgery. (C) Left ventriculography revealed compression of the true lumen of the ascending aorta from the false lumen of the ascending aorta during the beat as shown in the right figure (white arrows). LMT, left main trunk.

**Figure 4:**
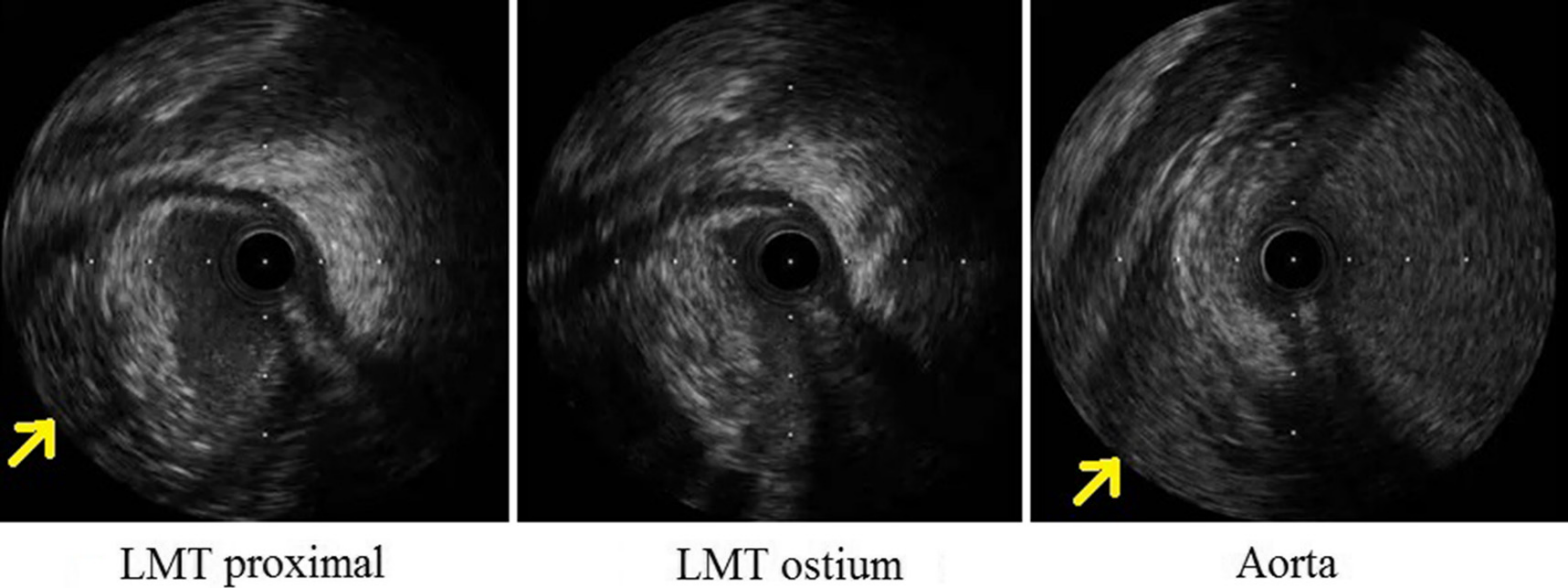
Intravascular ultrasound imaging. Intravascular ultrasound imaging revealed the false lumen at LMT proximal (note the yellow arrow, as shown in the left figure), which compressed the true lumen of LMT ostium (the midfigure). The false lumen was also observed in the ascending aorta (note the yellow arrow, as shown in the right figure). LMT, left main trunk.

A 45‐year‐old male presented at the emergency department complaining of sudden chest pain and dyspnea. He was transported and admitted to our hospital after 26 min of symptom onset. He had hypertension, dyslipidemia, diabetes mellitus, sleep apnea syndrome, and obesity as his medical history. He had no allergies and had no familial history of coronary artery disease. His Glasgow coma scale was 15 (E4V5M6), and his other vital signs on admission were as follows: blood pressure: 160/113 mmHg; heart rate: 74 beats/min; body temperature: 36.1°C (96.98°F); and pulse oximetry oxygen saturation (SpO_2_): 98% on room air. He was 173 cm tall and weighed 105 kg (body mass index was 35.1). A chest examination revealed normal heart and breath sounds. No leg edema was observed. Additional physical examinations revealed no abnormalities. Immediately after admission, electrocardiography (ECG) findings revealed normal sinus rhythm with narrow QRS duration, ST‐segment elevation in lead aVR, and ST‐segment depression in leads I, II, III, aVL, aVF, and V4‐6 (Fig. 1). Echocardiography also revealed broad anterolateral akinesis without aortic regurgitation, pericardial effusion, or definite aortic flap. The diameter of the aortic root was 45 mm (Fig. 1). As his chest pain had frequent improvements and exacerbations in a short period, we suspected not only the presence of acute coronary syndrome (ACS) of the culprit lesion with LMT, but also Stanford type‐A AAD involving LMT compression. As plain computed tomography (CT) imaging demonstrated no definitive signs of AAD with 42 mm in the shortest transverse diameter of the aortic root (Fig. 2), he underwent emergency coronary angiography (CAG) soon after the plain CT scan. Because there was no information about his renal function at the time pending the outcome of the blood test on admission and we were concerned about the possibility of the presence of ACS of the culprit lesion with LMT, he did not undergo contrast‐enhanced CT scan before CAG. Following some difficulties in manipulating the catheters, CAG revealed 75% stenosis of the LMT ostium (Fig. 3A). Intravascular ultrasound imaging (IVUS) revealed compression of the LMT ostium from the outside of the true lumen (Fig. 4). The vessel size of the LMT ostium was 4.9 mm × 4.7 mm by IVUS, and the lumen size of the LMT ostium was changed from 3.9 mm × 3.5 mm to 1.6 mm × 3.4 mm by IVUS according to LMT compression from the outside of the true lumen. As the patient was in shock state with respiratory failure requiring inotropic agents and intubation at the catheterization laboratory, a bare‐metal stent (3.5 mm × 9 mm) was implanted into the LMT ostium, 2–3 mm in the aorta, as a bridge to surgery with low pressure (nine atmosphere) so that a surgeon could pull out the stent during surgery (Fig. 3B). Antiplatelet agents were not administered in order to help minimize perioperative bleeding complications. A total 5000‐U amount of heparin was given during percutaneous coronary intervention (PCI). His hemodynamics were stabilized, and echocardiography revealed normal left ventricular function after PCI. Left ventriculography revealed compression of the true lumen of the ascending aorta from the false lumen of the ascending aorta during heart beats (Fig. 3C). Then, after PCI, he underwent contrast‐enhanced CT imaging, which indicated Stanford type‐A AAD (Fig. 2). He was safely transferred to another hospital to undergo surgery after three hours of stenting. He underwent hemiarch replacement with coronary artery bypass grafting (saphenous vein graft [SVG] to left anterior descending artery, and SVG to left circumflex artery). During the surgery, the stent was pulled out from the LMT ostium. He was discharged from the hospital to home on day 27.

## Discussion

Half of cases have ST‐T abnormalities even in the case of Stanford type‐A AAD 5, and ST‐segment elevation in lead aVR is a poor prognostic marker even in case of Stanford type‐A AAD 6; however; it is difficult to distinguish ACS due to the culprit lesion of LMT and Stanford type‐A AAD involving LMT compression by ECG. In addition, though aortic regurgitation, pericardial effusion, or aortic flap by echocardiography indicates the possibility of Stanford type‐A AAD, it is important to note that these findings are not always recognized in cases of Stanford type‐A AAD. Whereas contrast‐enhanced CT imaging is the best method to diagnose AAD, it takes some time to definitively do so, resulting in potentially fatal time loss if it is a case of ACS due to the culprit lesion of LMT.

As there was no ST‐segment elevation or wide QRS duration in this patient and we suspected Stanford type‐A AAD involving LMT compression based on the presence of his chest pain with frequent improvements and exacerbations, the patient underwent only plain CT imaging first in this case. Because plain CT imaging could not detect definite AAD, it was difficult for us to diagnose the patient with Stanford type‐A AAD involving LMT compression at that time. Even so, as we suspected Stanford type‐A AAD involving LMT compression, antiplatelet agents were not administered. Finally, as CAG and IVUS strongly indicated Stanford type‐A AAD involving LMT compression, a bare‐metal stent was implanted into the LMT ostium as a bridge to surgery with only a total administration of 5000‐U of heparin in this case. Even though there are some reports of LMT stenting as a bridge to surgery in patients with Stanford type‐A AAD 3, 7, 8, 9, 10, 11, 12, 13, 14, to the best of our knowledge, this is the first report of LMT stenting as a bridge to surgery in patients with Stanford type‐A AAD without the concomitant use of antiplatelet agents to minimize perioperative bleeding complications. In this case, the patient was safely transferred to another hospital to undergo surgery after three hours of stenting only having a total 5000‐U of heparin. Thrombogenic activity at coronary arteries may be low in patients with Stanford type‐A AAD as compared with those with ACS.

## Conclusions

In conclusion, in cases involving unstable hemodynamics in patients with Stanford type‐A AAD involving LMT compression, LMT stenting without antiplatelet agents may be a good option as a bridge to surgery.

## Consent for Publication

The patient and his family have given their informed consent for this case report to be published.

## Authorship

MG: was a major contributor in writing the manuscript. TE, KK, and KT: reviewed the final manuscript. All authors take responsibility for its integrity and have read and agreed to the manuscript as written.

## Conflicts of Interest

The authors declare that they have no competing interests.
